# Use of proteomics to identify mechanisms of hepatocellular carcinoma with the CYP2D6*10 polymorphism and identification of ANGPTL6 as a new diagnostic and prognostic biomarker

**DOI:** 10.1186/s12967-021-03038-3

**Published:** 2021-08-19

**Authors:** Guiming Hu, Fei Gao, Guanzhe Wang, Yan Fang, Yuanyuan Guo, Jun Zhou, Yuhan Gu, Cunzhen Zhang, Na Gao, Qiang Wen, Hailing Qiao

**Affiliations:** 1 Institute of Clinical Pharmacology, Zhengzhou University, Zhengzhou, 450052 Henan China; 2 Affiliated People’s Hospital of Zhengzhou University, Zhengzhou, China

**Keywords:** Polymorphisms, CYP2D6, Hepatocellular carcinoma, ANGPTL6, Proteomics

## Abstract

**Background:**

Although an association between the cytochrome P4502D6 (CYP2D6) *10 (100C>T) polymorphism and hepatocellular carcinoma (HCC) is known, the mechanism remains unclear. Here we aimed to explore mechanisms of CYP2D6*10 (100C>T) polymorphism conferring to HCC, and screen markers for HCC.

**Methods:**

Label-free global proteome profiling with 34 normal livers and peritumor tissue from 61 HCC patients was performed, and angiopoietin-like protein-6 (ANGPTL6) was evaluated in 2 liver samples validation cohorts and 2 blood specimens validation cohorts.

**Results:**

We found a significantly decreased frequency of TT in HCC patients which reduced HCC susceptibility by 69.2% and was accompanied by lowered enzymatic activity for CYP2D6. Proteomic analysis revealed 1342 differentially expressed proteins (DEPs) that were associated with HCC and 88 DEPs were identified as 100 TT-related proteins, likely underlying the susceptibility to HCC. Twenty-two upregulated DEPs and 66 downregulated DEPs were mainly related to lipid metabolism and the extracellular matrix, respectively. High ANGPTL6 was associated with a higher risk to HCC and worse prognosis. ANGPTL6 was both an independent risk factor and an independent prognostic factor for HCC and exhibited strong potential for predicting HCC occurrence, with comparable AUC values and higher sensitivity compared with alpha-fetoprotein.

**Conclusions:**

The TT genotype-associated decreased risk of HCC appears to be related to lowered CYP2D6 activity and altered protein expression in the tumor microenvironment, and ANGPTL6 is a promising new diagnostic and prognostic biomarker for HCC. Our findings reveal new mechanistic insights for polymorphisms related to HCC risk and provide avenues for screening for HCC.

**Supplementary Information:**

The online version contains supplementary material available at 10.1186/s12967-021-03038-3.

## Background

Numerous studies have reported the important role of gene polymorphisms in the development of numerous diseases [1–5]. However, there is still a lack of underlying mechanisms for these polymorphisms. Indeed, phenotypic changes related to diseases caused by genotypic changes involve numerous proteins and complex processes, making it difficult to determine mechanisms. However, currently available mass-spectrometry-based proteomics, which had evolved as the preferred method for the analysis of complex proteomes, provided unprecedented opportunities to elucidate the mechanisms by which gene polymorphisms lead to cancer.

Hepatocellular carcinoma (HCC), which is characterized by high morbidity and mortality rates, is the most common type of liver cancer and accounts for 90% of cases [6]. Hepatocarcinogenesis is well known to be associated with numerous genetic polymorphisms that are involved in its complex and multi-factorial development and contribute to malignant transformation [7–10]. To date, no effective intervention methods for HCC are based on genetics. Therefore, discovery of new genetic polymorphisms in HCC and exploration of their mechanisms might provide new approaches for identifying novel therapeutic targets and designing new or personalized treatment for HCC.

Cytochrome P450 2D6 (CYP2D6), is one of the most investigated CYPs in relation to its highly complicated polymorphisms, with 144 single nucleotide polymorphisms (SNPs) reported [11], resulting in remarkable differences in the activity of this enzyme [12, 13]. In recent years CYP2D6 has gained increasing attention on those polymorphisms that are related disease susceptibility, due to altered metabolism of its metabolically abundant substrates including approximately 25% of therapeutic drugs (about 223 drugs) and known and potential carcinogens such as nitrosamine, nicotine, and cotinine found in tobacco [14]. An increasing evidence suggest that CYP2D6 polymorphisms are related to susceptibility to numerous diseases, including autoimmune diseases [15], Alzheimer's disease [16] and Parkinson's disease [17]. It has been demonstrated that the frequency of CYP2D6-inactivating mutations is significantly lower among liver cancer patients than in healthy controls and cirrhotic subjects who did not develop liver cancer [18]. Moreover, CYP2D6 poor metabolizer genotypes were reported significantly more frequent in healthy subjects and carriers than in hepatitis/cirrhosis and HCC patients [19]. Although association between CYP2D6 gene polymorphisms and disease risk had been investigated in several studies, it is still unclear how CYP2D6 polymorphisms confer susceptibility to HCC.

Our previous study systematically assessed changes in 10 major CYP450 enzymes in HCC patients [20] and found that differences in CYP activity between physiological and pathological states were associated with increased risk for hepatofibrosis [21] and hepatocarcinogenesis [1, 22, 23]. CYP2D6*10 (100C>T) has been reported to be most closely associated with significantly altered metabolic activity and susceptibility to HCC, both in our and other studies [24–26]. However, the underlying mechanism by which this 100C>T mutation impacts hepatocarcinogenesis is not established. Thus, to address this problem, we performed label-free global proteome profiling with 34 normal livers and peritumors of 61 HCC patients to elucidate changes in the tumor microenvironment (TME) related to the CYP2D6*10 polymorphism. In addition, ANGPTL6 as a potential diagnosis and prognostic biomarker of HCC was evaluated in the above liver tissues and validated in the GEO database, another set of liver samples, and serum samples of 23 normal healthy controls and 42 HCC patients.

## Methods

### Study design

**Fig. 1:**
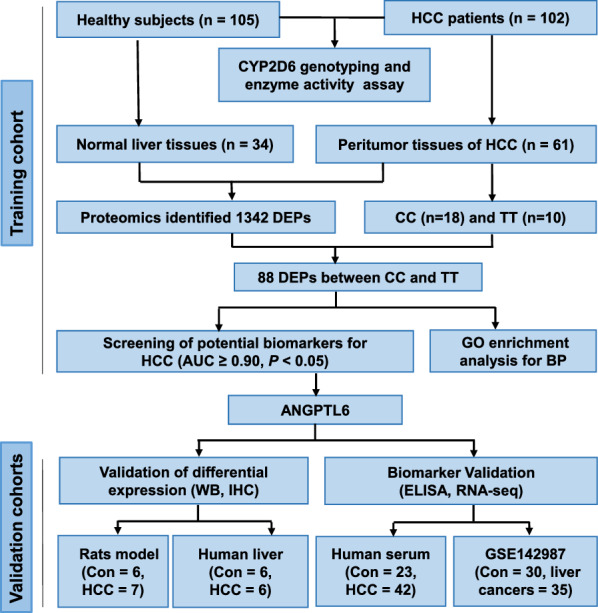
Workflow of the study. Clarification of mechanisms of HCC with CYP2D6*10 polymorphisms and screening of potential biomarkers using label-free global proteome analysis. CYP2D6, cytochrome P4502D6. CC and TT refer to CYP2D6*10 CC or TT genotype patients. ANGPTL6, angiopoietin‐like 6. AUC, area under the curve. BP, biological processes. GO, gene ontology. Con, control. RNA-seq, RNA sequencing. HCC, hepatocellular carcinoma. ELISA, enzyme-linked immunosorbent assay. WB, western blotting. IHC, immunohistochemistry

The study included a training cohort and four independent validation cohorts. The workflow of our study was summarized in Fig. 1. In the training cohort 105 healthy subjects and 102 HCC patients who had undergone hepatic surgery were included. Human and rat liver tissues, paraffin specimens, human serum samples and GEO database (GSE142987) constituted the validation cohorts.

### Human liver samples

Normal liver samples were from hepatic haemangioma patients with normal liver function and histologic appearance. Peritumor liver specimens were obtained about 2 cm distant from the tumor tissues from HCC patients without tumor radiotherapy, chemotherapy or targeted drug therapy before surgery. All liver specimens were stored in liquid nitrogen within 30 min of resection and until use. The data were recorded via an inpatient case record, including gender, age, laboratory tests such as aspartate amino transferase (AST), alanine aminotransferase (ALT), gamma-glutamyl transferase (GGT), alkaline phosphatase (ALP), platelets (PLT), alpha‐fetoprotein (AFP), bilirubin and so on. All 207 liver tissue samples were collected at The First Affiliated Hospital of Zhengzhou University, Henan Provincial People's Hospital and Henan Provincial Tumor Hospital (Henan, China); demographic characteristics are described in our previous research [24]. Paraffin samples of human livers included 6 normal livers, 6 peritumor tissues and tumor cores of HCC patients derived from the Second Affiliated Hospital of Zhengzhou University (Henan, China).

### Blood samples

Serum samples were drawn at 6:00–8:00 AM after an overnight fast from a validation cohort that included 27 healthy controls and 42 HCC patients from The First Affiliated Hospital of Zhengzhou University. A single venous blood sample (3–5 mL) was taken from each participant under aseptic conditions. Serum was separated by centrifugation and stored at − 20 °C, avoiding repeated freeze–thaw cycles prior to analysis.

### Genotypes of CYP2D6*10 (100C>*T)*

Genomic DNA was isolated from human liver tissues using a genomic DNA purification kit (QIAGEN Translational Medicine Co., Ltd, China). Genotyping of *CYP2D6 100C*>*T* of all participants was determined by Polymerase Chain Reaction (PCR) sequencing according to our previous study [25].

### Determination of enzyme activity of CYP2D6

Enzymic activity of CYP2D6 in human liver microtomes (HLMs) of 105 healthy subjects and 102 HCC patients was measured by metabolism of dextromethorphan (National Institute for the Food and Drug Control), a probe drug for CYP2D6. HLMs were prepared by hypothermal differential centrifugation. Briefly, incubation mixtures contained HLMs (0.2 mg/mL protein), 100 mM phosphate buffer (pH 7.4), different concentrations of substrate and 1 mM NADPH. The mixture was pre-incubated for 5 min at 37 °C. Optimal incubation time was 20 min for dextromethorphan *O*-demethylation. For biotransformation, 0.625–960 μM dextromethorphan was added. Reactions were terminated by adding 20 μL ice-cold acetonitrile; substrate metabolite was determined by high performance liquid chromatography-fluorescence detection (HPLC-FLD). The values of the Michaelis constant (K_m_) and V_max_ were determined by nonlinear regression analysis using GraphPad Prism 8.03 software. The intrinsic clearance (CL_int_) was calculated from the ratio of V_max_ to K_m_.

### Proteomics and data analysis

Proteomic analysis of 34 normal livers and peritumors of 61 HCC patients was performed by the State Key Laboratory of Proteomics, Beijing Proteome Research Center, (Beijing, China). Briefly, proteins were prepared by means of in-solution or in-gel digestion and subjected to liquid chromatography tandem mass spectrometry analysis (Q-Exactive HF LC–MS/MS). Raw data from mass spectrometry were analyzed against the human UniProt protein sequence database (version 20140922, 20,193 sequences) with MaxQuant software31 (version 1.5.3.8). The false discovery rate (FDR) for peptides was set to < 1%. The maximum mass error of the primary ion and product ion scans for the initial mass spectrometry were set to 20 ppm and 0.5 Da, respectively. The corrected error of the precursor ion was set to 5 ppm. A 6947 × 95 protein expression matrix was obtained from the 3.2.4 MaxQuant result file. Intensity-based absolute protein quantification (iBAQ) based on peak intensity was used to determine protein expression levels. The R/Bioconductor package limma v.3.24.15 34 was used to identify differentially expressed proteins (DEPs) between normal liver tissues and peritumor tissues or between CC and TT genotype groups. Proteins detected in < 50% of samples were excluded, and missing values were replaced with half the minimal value for each protein. Further details of the procedure for digesting proteins into peptides and proteomic analysis are described elsewhere [27].

### Functional enrichment analysis

Gene ontology (GO) analysis was performed to determine the potential biological functions of ANGPTL6 and were carried out using Enrich GO function in the R package “clusterProfiler”. GO analysis was according to the threshold of *P* < 0.05 and q < 0.05.

### Validation of Angiopoietin-like protein-6 (ANGPTL6) in liver tissues

Expression of ANGPTL6 in livers from 6 normal samples and 6 tumor-extrinsic tissues of HCC patients was determined by western blotting (WB) with primary anti-ANGPTL6 antibody (GTX118035, diluted at 1:1000). Further validation of ANGPTL6 in paraffin samples including 6 human normal livers and 6 tumor-extrinsic tissues of HCC patients was performed by immunohistochemistry (IHC) using anti-ANGPTL6 (GTX118035) antibody at 1:500 dilution. Antigen Retrieval: Trilogy™ (EDTA based, pH 8.0) buffer, 15 min.

### Allograft transplantation model

Sprague Dawley (SD) rats (180–220 g), purchased from ZhengZhou University, were anesthetized with 300 mg/kg chloral hydrate by intraperitoneal injection and then placed in a supine position on the operating table. To expose the left lobe of the liver, a 1.5 cm longitudinal incision was made below the xiphoid process. Walker256 cells (1.5 × 10^6^) in 50 μL RPMI-1640 medium (GIBCO RPMI 1640, HCC model group, n = 7) or 50 μL serum-free RPMI-1640 medium (Normal control group, n = 6) were slowly injected into the left liver lobe using a microinjector at a 30-degree angle. After allowing 3 min for the cells to disperse into the liver tissue, the skin was sutured and the surgical incision disinfected. The rats were kept in a Specific Pathogen Free (SPF) warm incubator until they had recovered from the anesthesia and then returned to animal room.

### Validation of ANGPTL6 in serum

The serum level of ANGPTL6 in 23 healthy controls and 42 HCC patients was determined by ELISA. A human ANGPTL6 enzyme-linked immunosorbent assay (ELISA) Kit (Catalog No. EK19979) was used to measure ANGPTL6 levels according to the manufacturer’s instructions. The analytical (linear) measurement range was 0.312–20 ng/mL with sensitivity of 0.077 ng/mL. The intra-assay coefficient of variation (CV) and inter-assay CV of ANGPTL6 were less than 10% and 15% respectively. In addition, quality controls were included in all measurements with the results within the expected range.

### Validation of ANGPTL6 in the GEO database

Serum mRNA levels of ANGPTL6 in 30 healthy controls and 35 liver cancers were obtained from the GEO database (GSE142987).

### Statistical analysis

Statistical analysis was performed using SPSS 23.0 software. Graphs were plotted by R software (Version 4.0.3) and Graphpad Prism 8.3.0 software. The data were summarized as mean ± SE. Differences between two independent groups were tested with the Wilcoxon Mann–Whitney test. Receiver operating characteristics (ROC) curves were constructed to assess sensitivity, specificity, and respective areas under the curves (AUCs). A two-tailed *P* value < 0.05 was regarded as statistically significant.

## Results

### Decreasing susceptibility to HCC is skewed in 100TT HCC patients and associated with metabolic activity of CYP2D6

**Fig. 2:**
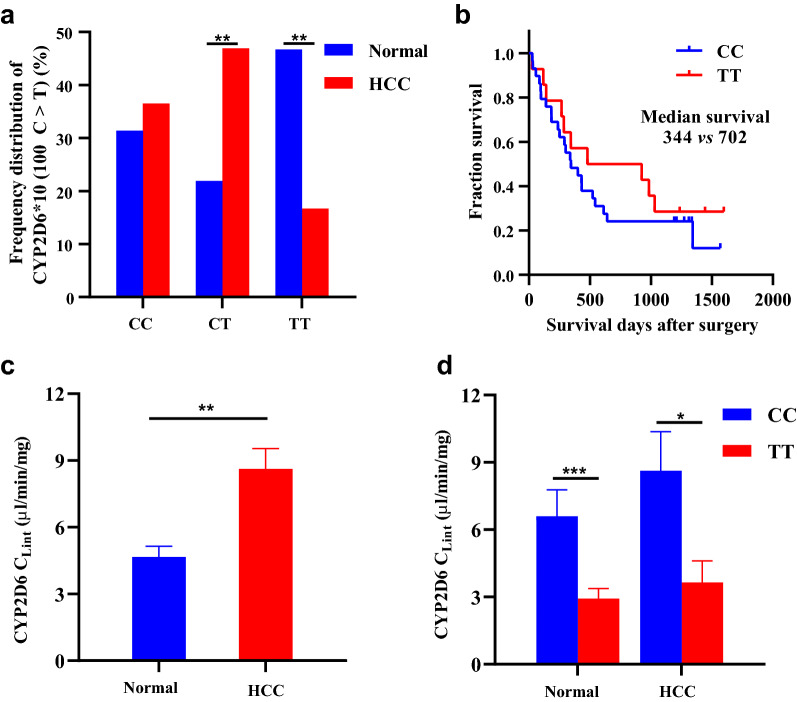
CYP2D6*10 genotype frequency and enzyme activity in healthy subjects and HCC patients. **a** The frequency distribution of CYP2D6*10 genotypes in HCC patients and a normal population. **P < 0.01 vs heathy subjects. **b** Kaplan–Meier curve for overall survival (OS) of HCC patients with the CC and TT genotype, *P*_log-rank_ = 0.365. **c** CYP2D6 activity of in peritumor liver tissues of HCC patients and normal liver tissues. **P < 0.01 vs heathy subjects. **d** CYP2D6 activity in subjects with the TT and CC genotype in healthy subjects and HCC patients. *P < 0.05, ***P < 0.001, TT group vs CC group. HCC, hepatocellular carcinoma; Healthy means healthy subjects. Data were expressed as mean ± SEM, *P* value was calculated by Mann–Whitney U test

A Chi-square test revealed that the study population is in Hardy–Weinberg equilibrium for the CYP2D6 gene (*P* > 0.05), which suggest that the genotype frequency distribution had a group representation (Additional file 1: Table S1). Significantly decreased frequency of TT was discovered in HCC patients compared with healthy subjects (Fig. 2a, 16.7% vs 46.7%, *p* < 0.01), as evidenced by the frequency of CC, CT and TT (6.5%, 46.5% and 16.7% respectively) in HCC patients and healthy subjects (31.4%, 21.9% and 46.7%, respectively) (Additional file 2: Table S2).

CYP2D6*10 polymorphisms were significantly associated with susceptibility to HCC, as the risk of HCC in individuals carrying the TT genotype decreased by 69.2% [(Odds Ratio (OR) = 0.308, 95% confidence interval (CI) 0.147–0.644, *P* = 0.002)] when compared with CC genotype carriers (Additional file 2: Table S2), suggesting that the TT genotype is a protective factor for HCC. The above findings were confirmed by the overall survival (OS) of HCC patients. A Kaplan–Meier survival curve revealed that patients with the TT genotype had longer OS than those with the CC genotype (median OS 702 vs 344 days, *P* = 0.365, Fig. 2b). The survival difference did not reach statistical significance, possibly due to the small number of cases. This conclusion was further substantiated by the biochemical data obtained in HCC patients. As shown in Table 1, platelets (PLT) and the albumin/globulin ratio (A/G) were higher in patients with the TT genotype (*P* = 0.022 and *P* = 0.020, respectively), while total bilirubin (TBIL), direct bilirubin (DBIL) and prothrombin time (PT) were lower when compared with individuals carrying the CC genotype (*P* = 0.009, *P* = 0.043, *P* = 0.043, respectively). There was no significant difference in distribution of gender and age between the two groups. Taken together, these results suggest that homozygous TT mutations may play an important role in the decreased susceptibility to HCC.

**Table 1:** The basic clinical characteristics of HCC patients with CC (n = 35) and TT (n = 16) genotype

Variables	CC n (%)	TT n (%)	*P*
Age (year)			0.091
≤ 50	13 (37.14)	10 (62.50)	
> 50	22 (62.86)	6 (37.50)	
Gender			0.370
Man	31 (88.57)	13 (81.25)	
Female	4 (11.43)	3 (18.75)	
PLT (× 10^9^/L)			0.022
< 180	23 (65.71)	5 (37.50)	
≥ 180	12 (34.29)	11 (62.50)	
ALT (U/L)			0.360
≤ 40	23 (65.71)	8 (50.00	
> 40	12 (34.29)	8 (50.00)	
AST (U/L)			0.179
≤ 40	18 (51.43)	5 (31.25)	
> 40	17 (48.57)	11 (68.75)	
Albumin/globulin			0.020
≤ 1.5	21 (60.00)	4 (33.33)	
> 1.5	14 (40.00)	12 (66.67)	
ALB (g/L)			0.355
≤ 70	18 (51.43)	6 (37.50)	
> 70	17 (48.57)	10 (62.50)	
TBIL (μmol/L)			0.009
≤ 17	20 (57.14)	15 (93.75)	
> 17	15 (42.86)	1 (6.67)	
DBIL (μmol/L)			0.043
≤ 7	18 (51.43)	13 (81.25)	
> 7	17 (48.57)	3 (18.75)	
ALP (U/L)			0.242
≤ 130	27 (77.14)	15 (93.75)	
> 130	8 (22.86)	1 (6.67)	
AFP (ng/mL)			0.246
≤ 300	17 (48.57)	5 (31.25)	
> 300	18 (51.43)	11 (68.75)	
PT (s)			0.043
≤ 14	26 (74.29)	16 (100.00)	
> 14	9 (25.71)	0 (0)	

*P* values were calculated from two‐side chi‐square test

AFP: alpha-fetoprotein; ALB: albumin; ALT: alanine amino transferase; ALP: alkaline phosphatase; AST: Aspartate amino transferase; DBIL: direct bilirubin; PLT: platelet; PT: prothrombin time; TBIL: total bilirubin

To uncover the underlying mechanism, we next assessed the effects of CYP2D6*10 polymorphisms on enzyme activity. As measured by dextromethorphan O-demethylation metabolism in human liver microsomes, the intrinsic clearance (Cl_int_) of CYP2D6 increased significantly in HCC patients compared with normal controls (Median: 6.86 μL/min/mg vs 3.49 μL/min/mg, *P* < 0.01, Fig. 2c). What’s more, lower metabolic activity was observed among TT genotype subjects when compared with CC genotype individuals both in healthy samples (Median: 1.775 μL/min/mg vs 5.079 μL/min/mg, *P* < 0.001) and in the HCC group (Median: 1.792 μL/min/mg vs 6.228 μL/min/mg, *P* = 0.045, Fig. 2d). Taken together, these results suggest that a significantly reduced frequency of TT genotype was associated with decreased HCC susceptibility, at least partially, probably by decreasing the metabolic activity of CYP2D6.

### Proteomic mechanisms of CYP2D6*10 polymorphisms that confer susceptibility to HCC

**Fig. 3:**
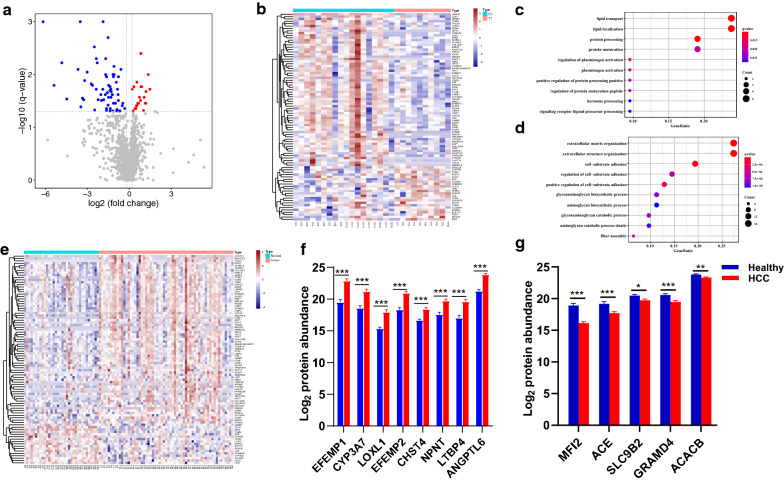
Proteomic mechanisms of CYP2D6*10 polymorphisms on susceptibility to HCC. **a** Volcano plot showing 22 upregulated and 66 downregulated proteins. **b** Total of 88 DEPs between CC and TT groups of HCC patients shown in heat map. GO enrichment analysis shows up-regulated (**c**) and down-regulated (**d**) proteins in the TT group. **e** Heat-map showing 88 statistically significant DEPs between normal individuals (n = 34) and HCC patients (n = 61). **f** Expression profiles of the top nine significantly up-regulated DEPs in peritumoral tissues of HCC. **g** Expression profiles of the top five significantly down-regulated DEPs in peritumoral tissues. Data were shown with mean ± SEM, *P < 0.05, **P < 0.01, ***P < 0.001 (M-U-test). DEPs, differentially expressed proteins; HCC, hepatocellular carcinoma

To investigate the mechanism by which the CYP2D6*10 mutation reduces HCC susceptibility, we performed proteomic analysis of liver tissues from 34 normal subjects and 61 HCC patients to identify HCC related proteins. A total of 1342 DEPs were identified. We then focused on 18 CC genotypes and 10 TT genotypes derived from the above HCC patients to identify DEPs by using a filter criterion of |log2-fold change (FC)|≥ 0.20 and *P* value < 0.05. As shown in the volcano plot (Fig. 3a) and heat-map (Fig. 3b), 88 statistically significant DEPs were identified, including 22 upregulated and 66 downregulated proteins (Additional file 3: Table S3).

The gene ontology (GO) term enrichment biological process analysis revealed that upregulated DEPs were significantly enriched in lipid transport, lipid localization, sterol metabolic process, lipid storage and cholesterol biosynthetic processes (Fig. 3c). Downregulated DEPs were mostly enriched in extracellular matrix organization, extracellular structure organization, and cell − substrate adhesion categories (Fig. 3d). Patients with the TT genotype had a lower risk of HCC which might be related to an altered tumor-extrinsic microenvironment. Subsequently, we investigated the differential expression of these CYP2D6*10-related DEPs in normal subjects and HCC patients. Surprisingly, an opposite trend further confirmed that the peritumoral microenvironment of these two genotypes was different. As shown in Fig. 3e, most of the up-regulated proteins (68.2%, 15 out of 22) in the TT genotype were lowly expressed, while the vast majority of downregulated proteins (97.0%, 64 out of 66) in this genotype were highly expressed in HCC (Additional file 4: Table S4). The significantly upregulated top nine DEPs—EFEMP1, CYP3A7, LOXL1, EFEMP2, MFI2, CHST4, CCL21, NPNT, LTBP4 and ANGPTL6, and downregulated top five DEPs—MIF2, ACE, SLC9B2, GRAMD4 and ACACB in the peritumoral microenvironment of HCC are shown in Fig. 3f and g. These results clearly demonstrate that patients with the TT genotype had lower risk of HCC that might result, at least in part, from an altered tumor-extrinsic microenvironment, perhaps by upregulating proteins related to lipid metabolism and downregulating proteins related to the extracellular matrix.

### Screening biomarkers for HCC based on proteomics

**Fig. 4:**
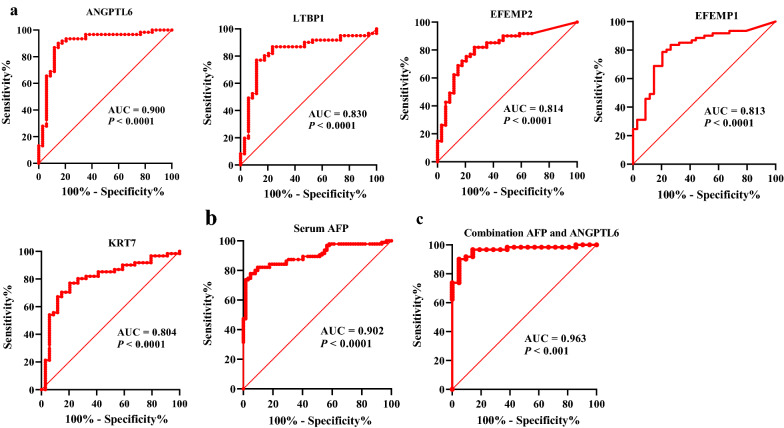
Diagnostic performance of the top five DEPs with AUC > 0.80 and serum AFP. **a** Receiver operating characteristic (ROC) curve shows the predictive power of the top 5 DEPs with AUC > 0.80. **b** ROC curve of serum AFP for HCC patients versus healthy subjects. **c** ROC curve of combination AFP and ANGPTL6. HCC, hepatocellular carcinoma, DEPs, differentially expressed proteins

A receiver operating characteristic (ROC) curve on the 100C >T-related DEPs revealed that 30 of the 88 proteins showed high ability to distinguish HCC patients from healthy subjects with a high AUC (> 0.70) (Additional file 5: Table S5). The top five proteins with the AUC > 0.80 were ANGPTL6 (AUC 0.900, sensitivity 91.8%, specificity 81.1%), LTBP1 (AUC 0.830, sensitivity 77.0%, specificity 88.2%), EFEMP2 (AUC 0.814, sensitivity 82.0%, specificity 73.5%), EFEMP1 (AUC 0.813, sensitivity 78.7%, specificity 79.4%) and KRT7 (AUC 0.804, sensitivity 77.0%, specificity 79.4%). The largest AUC was for ANGPTL6, which was comparable to serum AFP with an AUC of 0.900 (Fig. 4a and b). Interestingly, the detection rate of AFP in normal liver tissues and peritumoral tissues of HCC was 2.94% (1/34) and 18.03% (11/61), respectively, which was much lower than those of the top five proteins. What’s more, the combination of ANGPTL6 and AFP increased the diagnostic accuracy to a great extent when compared with ANGPTL6 or AFP alone, yielding an AUC of 0.963 and a sensitivity of 90.2% for HCC (Fig. 4c). Collectively, the above results show that in the parallel study the top five proteins, particularly ANGPTL6, exhibited a significantly high diagnostic performance whether alone or in combination with AFP in discriminating HCC from the healthy controls.

**Fig. 5:**
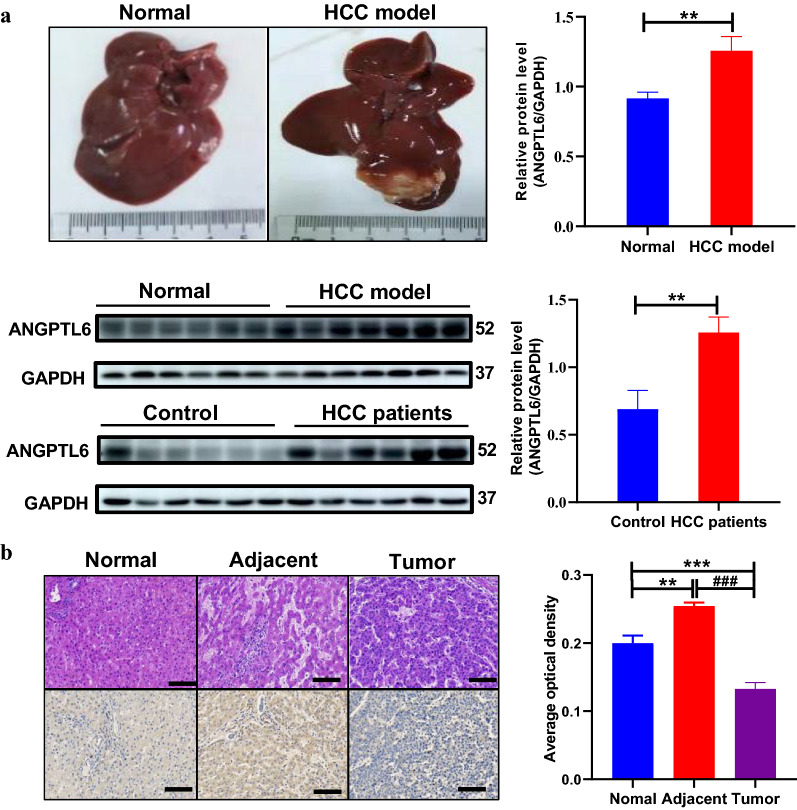
Verification of ANGPTL6 in animals and human samples. **a** General picture and western blot analysis of ANGPTL6 in Sprague Dawley rat model and human samples. **b** Representative images of H&E staining (top) and immunohistochemistry staining for ANGPTL6 (bottom), from human normal liver tissue, HCC tumor, and its peritumoral tissues. Scale bar, 100 μm. Data were expressed as mean with SEM. Statistical analysis was performed by Mann–Whitney test. **P < 0.01 vs Normal or Control, ^###^*P* < 0.001 vs adjacent. HCC, hepatocellular carcinoma

Among the 88 newly identified CYP2D6*10 related DEPs, we focused on ANGPTL6, a secretory protein, due to its high expression in peritumoral tissues and highest AUC for predicting HCC. In line with the proteomic results, ANGPTL6 was higher in peritumor specimens than normal livers both in human liver samples and in a rat HCC model (*P* < 0.01, respectively, Fig. 5a). Meanwhile, paraffin specimen analysis further confirmed these results and demonstrated almost no expression of ANGPTL6 in tumor cores (Fig. 5b).

### High ANGPTL6 is a risk factor for HCC and associated with poor prognosis

**Fig. 6:**
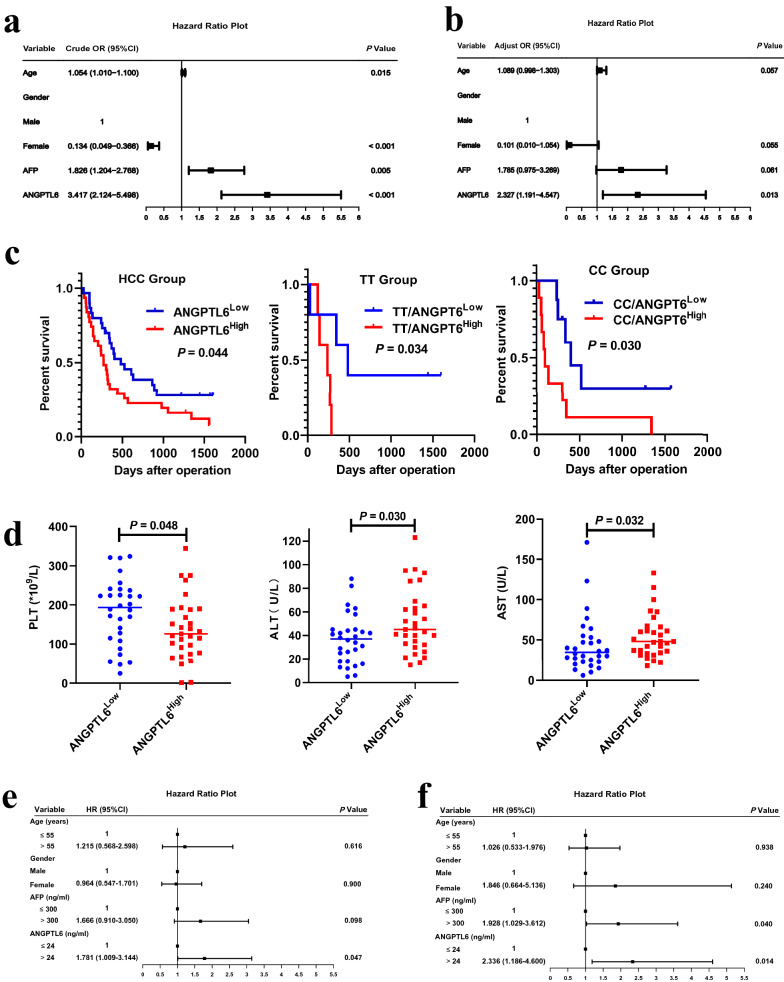
Clinical significance of ANGPTL6. Forrest plot of the univariate (**a**) and multivariate (**b**) logistic regression analysis. **c** High expression of ANGPTL6 conferring to a poor prognosis. **d** ANGPTL6 correlated negatively with PLT and positively with ALT and AST. Univariate (**e**) and (**f)** multivariate Cox regression analysis for OS. AFP, alpha-fetoprotein. PLT, platelet. ALT, alanine aminotransferase. AST, aspartate aminotransferase. OS, overall survival

To further elucidate the diagnostic potential of ANGPTL6, univariate and multivariate logistic regression analysis was performed on the training set. As shown in the forest maps (Fig. 6a), the univariate logistic regression analysis revealed that a high level of ANGPTL6 was associated with high risk for HCC [Odds ratio (OR): 3.417, 95% confidence interval (CI) 2.124–5.498; *P* < 0.001]. Multivariate logistic analysis showed that ANGPTL6 was an independent risk factor for HCC (OR: 2.327, 95%CI 1.191–4.547, *P* = 0.013, Fig. 6b). Collectively, these data strongly suggest that up-regulation of ANGPTL6 in peritumoral tissues of HCC might contribute to the pathogenesis of HCC.

Kaplan–Meier survival analysis indicated that higher ANGPTL6 in patients with HCC was associated with worse overall survival (OS), suggesting an important role of ANGPTL6 in HCC progression (Fig. 6c). Moreover, compared with the low expression group, patients with higher ANGPTL6 had lower PTL (*P* = 0.048), higher alanine aminotransferase (ALT, *P* = 0.030) and aspartate aminotransferase (AST, *P* = 0.032 Fig. 6d). Interestingly, older patients had a significantly higher level of ANGPTL6 than young patients (Additional file 6: Figure S1A). Serum levels of gamma-glutamyl transpeptidase (GGT) and PT showed an increasing trend in the high ANGPTL6 groups (Additional file 6: Figure S1B and S1C). What’s more, ANGPTL6 seemed to be higher in CC genotype patients than TT group (Additional file 6: Figure S1D). Univariate analysis revealed that high expression of ANGPTL6 showed a significant correlation with poor OS [hazard ratio (HR): 1.781, 95% confidence interval (CI) 1.009–3.144; *P* = 0.047, Fig. 6e]. Multivariate Cox analysis showed that ANGPTL6 was an independent prognostic factor for HCC (OR: 2.336, 95%CI 1.186–4.600, *P* = 0.014, Fig. 6f). Taken together, these data strongly suggest that high expression of ANGPTL6 is an independent prognostic indicator that correlates with poor prognosis.

### Validation ANGPTL6 as diagnostic marker in serum

**Fig. 7:**
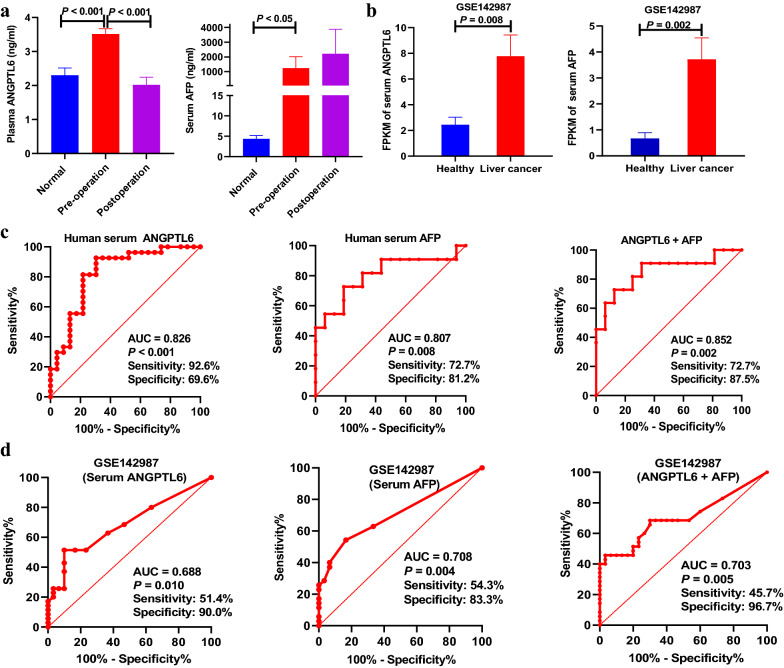
Identification of serum ANGPTL6 as a sensitive biomarker for HCC. ANGPTL6 and AFP protein level (**a**) and mRNA level (**b**) in plasma or serum. ROC curve of serum ANGPTL6, AFP alone or combination of ANGPTL6 and AFP for diagnosis of HCC in our validation set (**c**) and GEO database (**d**)

Serum ANGPTL6 and AFP from another validation cohort with 27 normal healthy volunteers and 42 HCC patients was determined to explore whether the serum level of ANGPTL6 can be used as an indicator for the diagnosis of HCC. There was a significant increase in serum ANGPTL6 and AFP levels in patients with HCC before surgery compared with healthy controls (Fig. 7a). Similar results were corroborated by the validation set obtained from the GEO database (GSE142987, Fig. 7b) in 30 healthy control and 35 liver cancers for mRNA expression levels. Surprisingly, the mean concentration of plasma ANGPTL6 before surgery was 3.51 (SD 0.83) ng/mL, and values dropped to 2.02 (SD 0.88) ng/mL (*P* < 0.001) after surgery; however, AFP was still high in patients seven days after undergoing hepatic surgery or receiving interventional therapy (Fig. 7a), suggesting that ANGPTL6 can monitor the curative effect dynamically.

We further assessed the accuracy of serum ANGPTL6 levels in the diagnosis of HCC. ROC curves showed that serum ANGPTL6 (AUC 0.826, sensitivity 92.6%, specificity 69.6%) had a comparable diagnostic value with AFP (AUC 0.807, sensitivity 72.7%, specificity 81.2%, Fig. 7c). The combination of ANGPTL6 with AFP provided better diagnostic performance of preclinical HCC before clinical diagnosis (AUC 0.852, sensitivity 72.7%, specificity 87.5%, Fig. 7c). Similar results were corroborated by the validation set obtained from the GEO database (GSE142987, AUC, 0.688 vs 0.708, Fig. 7d). These results conclusively demonstrate that serum ANGPTL6 is an indicator for the diagnosis of HCC.

## Discussion

In the present study, we demonstrated that CYP2D6 polymorphisms alter the susceptibility to HCC by decreasing the enzyme’s activity. Furthermore, based on proteomic analysis, we demonstrated a significant difference in the TME between CC- and TT-genotype patients that decreases the susceptibility to HCC in TT carriers, at least partly, by upregulating proteins related to lipid metabolism and downregulating proteins related to the extracellular matrix. Our current findings also reveal ANGPTL6 as a promising diagnostic and prognostic biomarker for HCC.

Relationships between genetic polymorphisms and cancer risk have been widely studied [28, 29]. However, most studies focused on polymorphisms and tumor susceptibility on the basis of epidemiologic studies, with the underlying mechanisms undiscovered. Recent advances in high-throughput proteomic technology provide expanded approaches into proteomic analyses with breadth and throughput not previously possible [30, 31]. Here, proteomic analysis was carried out to uncover the mechanisms of susceptibility to HCC underlying a CYP2D6 polymorphism. Our findings provide a unique insight into possible early intervention for HCC, especially in patients with cirrhosis.

In this study we found a significantly decreased frequency of 100TT-related genotypes with decreased susceptibility to HCC, consistent with previous studies that reported that CYP2D6 poor metabolizer (PM) genotypes or inactivating alleles were significantly more frequent in healthy subjects and carriers than in patients with hepatitis/cirrhosis and HCC [18, 19]. We speculated that the decrease in HCC susceptibility in TT genotypes may be partly explained by decreased CYP2D6 activity, due to its important role in activation of procarcinogens such as nitrosamine, nicotine, cotinine [14, 32].

Subsequently, based on proteomics, we identified a total of 1342 DEPs related to HCC, among which, 88 DEPs constitute a different TME between CC and TT and susceptibility to HCC. These findings could be explained by upregulated and downregulated DEPs that were mainly involved in lipid metabolism and the extracellular matrix, respectively, two biologic processes closely related with HCC development [33, 34]. Moreover, most of downregulated DEPs, which are involved in extracellular matrix organization, extracellular structure organization, and cell − substrate adhesion were upregulated in HCC. These findings together further validated that the TT variant was a protective factor in the occurrence of HCC and confirmed that an altered TME played a key role in polymorphisms related to susceptibility to HCC.

One of the main reasons for the high mortality rate of HCC is the lack of sensitive and reliable early diagnostic markers for screening HCC at early stage. Alpha fetoprotein (AFP) is the only currently available blood test commonly regarded as a characteristic biomarker for screening, detection and surveillance of HCC [35]. Unfortunately, the clinical utility is limited because of low sensitivity. Therefore, it is urgent to find novel effective predictive biomarkers with early diagnostic value for HCC. ANGPTL-6, also called angiopoietin-related growth factor protein 5 (ARP5), initially known as a circulating proangiogenic factor mainly secreted from the liver [36–38], represents an attractive therapeutic target for anti-tumor therapy [39]. In the current study, ANGPLT6 is dramatically increased both in peritumor tissues and serum and enriched in the extracellular matrix of HCC, suggesting an important role in the initiation and development of HCC. Upregulated ANGPTL6 expression resulted in the proliferation of undifferentiated glioblastoma cells [40], and knockdown of ANGPLT6 in vivo significantly inhibited AFP-producing gastric cancer by regulating endothelial cell migration and tube formation [39]. The diagnostic accuracy of ANGPTL6 was comparable to AFP; the AUC values for the two biomarkers were 0.900 and 0.902, respectively, while the sensitivity of ANGPTL6 was markedly superior to AFP (91.8% vs 73.8%). Regrettably, the number of serum samples in our study was too small to allow stratified analyses. In particular, some AFP data were missing, possibly leading to bias in the results, such as combination of ANGTPL6 and AFP not showing significant advantages over a single biomarker. A larger sample size is needed to confirm the clinical application value of ANGPTL6.

Hematological results showed high ANGPTL6 levels were associated with lower PTL and higher ALT and AST levels, and conferred a shorter OS. Univariate and multivariate Cox proportional hazards regression analysis revealed that high expression of ANGPTL6 was an independent prognostic indicator that correlated with poor prognosis. As a consequence, our current findings highlight ANGPTL6 as a promising diagnostic and prognostic biomarker for HCC.

## Conclusions

In summary, our findings support the mechanism by which CYP2D6*10 (100C>T) polymorphisms confer susceptibility to HCC by altering CYP2D6 activity and TME, which contributes to hepatocarcinogenesis. We further highlight ANGPTL6 as a promising diagnostic and prognostic biomarker for HCC. Our findings open new mechanistic insights for polymorphisms related HCC risk and provide avenues for clinical screening for HCC.

## Supplementary Information

**Additional file 1: Table S1.** Hardy–Weinberg Equilibrium (HWE).

**Additional file 2: Table S2.** The genotype frequency of CYP2D6*10 (100 C>T) in healthy volunteers (n = 105) and HCC patients (n = 96).

**Additional file 3: Table S3.** Compared with CC groups, the 22 upregulated and 66 downregulated DEPs in TT groups based on proteomics analysis.

**Additional file 4: Table S4.** Compared with healthy subjects, the 17 upregulated and 71 downregulated DEPs in HCC groups based on proteomics analysis.

**Additional file 5: Table S5.** DEPs with the area under the ROC curve (AUC) > 7.0.

**Additional file 6: Figure S1.** ANGPTL6 has clinical significance. **a** High ANGPTL6 expression was more common in the elderly group. Serum levels of GGT (**b**) and PT (**c**) show an increasing trend in the high ANGPTL6 groups. **d** ANGPTL6 in TT and CC group. HCC, hepatocellular carcinoma. GGT, gamma-glutamyl transpeptidase. PT, prothrombin time.

## Data Availability

All the data generated and analyzed during this study are included in the manuscript and the additional materials.

## Abbreviations

AFP: Alpha-fetoprotein
A/G: Albumin/globulin ratio
ALB: Albumin
ALT: Alanine aminotransferase
ANGPTL6: Angiopoietin-like protein-6
AST: Aspartate aminotransferase
AUC: Area under the curve
CI: Confidence interval
Cl_int_: Intrinsic clearance
CYP2D6: Cytochrome P4502D6
DBIL: Direct bilirubin
DEPs: Differentially expressed proteins
HCC: Hepatocellular carcinoma
HR: Hazard ratio
OR: Odds ratio
OS: Overall survival
PTL: Platelets
SD: Sprague Dawley
SNPs: Single nucleotide polymorphisms
TBIL: Total bilirubin
TME: Tumor microenvironment
ROC: Receiver operator characteristics curve
