# Supplementary material for: Use of proteomics to identify mechanisms of hepatocellular carcinoma with the CYP2D6*10 polymorphism and identification of ANGPTL6 as a new diagnostic and prognostic biomarker
Source: J Transl Med. 2021 Aug 19;19:359. doi: 10.1186/s12967-021-03038-3 (PMC8375140; doi:10.1186/s12967-021-03038-3)
Supplement: Supplementary file 1 — Additional file 1: Table S1. Hardy–Weinberg Equilibrium (HWE). [file 12967_2021_3038_MOESM1_ESM.docx]

**Table S1 Hardy–Weinberg Equilibrium (HWE)**

| Groups | Frequency | No. | Genotype frequency | | | Allele frequency | |
| --- | --- | --- | --- | --- | --- | --- | --- |
|  |  |  | **CC** | **CT** | **TT** | **C** | **T** |
| Control | **Actual frequency** | **33** | **12(0.363)** | **11(0.333)** | **10(0.303)** | **35(0.530)** | **31(0.470)** |
|  | **Theoretical frequency** | **33** | **9.27(0.281)** | **8.22(0.249)** | **7.29(0.221)** |  |  |
| HCC | **Actual frequency** | **57** | **18(0.316)** | **29(0.509)** | **10(0.175)** | **65(0.570)** | **49(0.430)** |
|  | **Theoretical frequency** | **57** | **18.53(0.325)** | **27.93(0.490)** | **10.54(0.185)** |  |  |

***X*^2^ = 2.21342, df = 2, *P* > 0.05**
