# Supplementary material for: Use of proteomics to identify mechanisms of hepatocellular carcinoma with the CYP2D6*10 polymorphism and identification of ANGPTL6 as a new diagnostic and prognostic biomarker
Source: J Transl Med. 2021 Aug 19;19:359. doi: 10.1186/s12967-021-03038-3 (PMC8375140; doi:10.1186/s12967-021-03038-3)
Supplement: Supplementary file 2 — Additional file 2: Table S2. The genotype frequency of CYP2D6*10 (100 C>T) in healthy volunteers (n = 105) and HCC patients (n = 96). [file 12967_2021_3038_MOESM2_ESM.docx]

**Table S2 The genotype frequency of *CYP2D6**10 (100 C>T) in healthy volunteers (n = 105) and HCC patients (n = 96).**

| Genotype | Healthy volunteers  n (%） | HCC patients  n (%) | *P* value | Crude OR (95% CI) | *P* value |
| --- | --- | --- | --- | --- | --- |
| CC | **33 (31.4)** | **35 (36.5)** | **< 0.001** | **1** |  |
| CT | **23 (21.9)** | **45 (46.9)** |  | **1.845 (0.924-3.684)** | **0.083** |
| TT | **49 (46.7)** | **16 (16.7)** |  | **0.308 (0.147-0.644)** | **0.002** |
| C | **89 (42.4)** | **115 (59.9)** | **< 0.001** | **1** |  |
| T | **121 (57.6)** | **77 (40.1)** |  | **0.492 (0.331-0.733)** | **< 0.001** |

OR, Odds ratio; 95% CI, 95% confidence interval; HCC, hepatocellular carcinoma.
