# Supplementary material for: Use of proteomics to identify mechanisms of hepatocellular carcinoma with the CYP2D6*10 polymorphism and identification of ANGPTL6 as a new diagnostic and prognostic biomarker
Source: J Transl Med. 2021 Aug 19;19:359. doi: 10.1186/s12967-021-03038-3 (PMC8375140; doi:10.1186/s12967-021-03038-3)
Supplement: Supplementary file 3 — Additional file 3: Table S3. Compared with CC groups, the 22 upregulated and 66 downregulated DEPs in TT groups based on proteomics analysis. [file 12967_2021_3038_MOESM3_ESM.docx]

**Table S3** **Compared with CC groups, the 22 upregulated and 66 downregulated DEPs in TT groups based on proteomics analysis**

| **Proteins** | **Genes** | **logFC** | ***P* value** | **Groups** |
| --- | --- | --- | --- | --- |
| **Melanotransferrin**  **Angiotensin-converting enzyme;Angiotensin-converting enzyme, soluble form**  **Mitochondrial sodium/hydrogen exchanger 9B2**  **Perilipin-4**  **GRAM domain-containing protein 4**  **Arf-GAP domain and FG repeat-containing protein 1**  **Ataxin-2-like protein**  **Exocyst complex component 3**  **Acetyl-CoA carboxylase 2;Biotin carboxylase**  **Lethal(2) giant larvae protein homolog 2**  **Beta-chimaerin**  **ERO1-like protein beta**  **Cytochrome P450 4V2**  **Integrin beta-3**  **Serine protease hepsin;Serine protease hepsin non-catalytic chain;Serine protease hepsin catalytic chain**  **Glycine dehydrogenase (decarboxylating), mitochondrial**  **Phospholipid-transporting ATPase IG**  **Peroxisomal targeting signal 1 receptor**  **Dehydrogenase/reductase SDR family member 1**  **Lanosterol synthase**  **NADH dehydrogenase [ubiquinone] 1 alpha subcomplex subunit 10, mitochondrial**  **Extended synaptotagmin-1** | **MFI2**  **ACE**  **SLC9B2**  **PLIN4**  **GRAMD4**  **AGFG1**  **ATXN2L**  **EXOC3**  **ACACB**  **LLGL2**  **CHN2**  **ERO1LB**  **CYP4V2**  **ITGB3**  **HPN**  **GLDC**  **ATP11C**  **PEX5**  **DHRS1**  **LSS**  **NDUFA10**  **ESYT1** | **1.474148447**  **1.353435326**  **1.193730074**  **1.181929463**  **1.135191309**  **1.057917524**  **1.014844133**  **0.889136413**  **0.829706717**  **0.82473838**  **0.799780757**  **0.72360326**  **0.661560924**  **0.605840216**  **0.544932679**  **0.535064853**  **0.507635277**  **0.466445382**  **0.404538374**  **0.331770144**  **0.277645921**  **0.209078227** | **0.019**  **0.01**  **0.021**  **0.035**  **0.035**  **0.027**  **0.048**  **0.017**  **0.017**  **0.004**  **0.031**  **0.027**  **0.035**  **0.035**  **0.014**  **0.039**  **0.039**  **0.044**  **0.044**  **0.017**  **0.049**  **0.019** | **up-regulated**  **up-regulated**  **up-regulated**  **up-regulated**  **up-regulated**  **up-regulated**  **up-regulated**  **up-regulated**  **up-regulated**  **up-regulated**  **up-regulated**  **up-regulated**  **up-regulated**  **up-regulated**  **up-regulated**  **up-regulated**  **up-regulated**  **up-regulated**  **up-regulated**  **up-regulated**  **up-regulated**  **up-regulated** |
| **Lamin-B1**  **Deoxynucleoside triphosphate triphosphohydrolase SAMHD1**  **Basement membrane-specific heparan sulfate proteoglycan core protein;Endorepellin;LG3 peptide**  **Vimentin**  **HLA class II histocompatibility antigen, DR alpha chain**  **Annexin A2;Putative annexin A2-like protein**  **Solute carrier family 35 member F6**  **Keratin, type II cytoskeletal 7**  **Filamin-A**  **Fibrillin-1**  **Phospholipid-transporting ATPase IC**  **Prolargin**  **Ig alpha-1 chain C region**  **Serine/threonine-protein phosphatase 6 regulatory ankyrin repeat subunit B**  **EMILIN-1**  **Nidogen-1**  **Laminin subunit alpha-5**  **Drebrin**  **Agrin;Agrin N-terminal 110 kDa subunit;Agrin C-terminal 110 kDa subunit;Agrin C-terminal 90 kDa fragment;Agrin C-terminal 22 kDa fragment**  **Aquaporin-1**  **Angiopoietin-related protein 6**  **Lymphocyte-specific protein 1**  **Glypican-6;Secreted glypican-6**  **von Willebrand factor A domain-containing protein 1**  **6-phosphofructo-2-kinase/fructose-2,6-bisphosphatase 2;6-phosphofructo-2-kinase;Fructose-2,6-bisphosphatase**  **Protein S100-A10**  **Thy-1 membrane glycoprotein**  **Granzyme K**  **Collagen alpha-1(XII) chain**  **Delta-sarcoglycan**  **Protein tyrosine phosphatase receptor type C-associated protein**  **Tumor-associated calcium signal transducer 2**  **Solute carrier family 12 member 2**  **Alpha-protein kinase 3**  **Thrombospondin type-1 domain-containing protein 4**  **Laminin subunit beta-1**  **Adipocyte enhancer-binding protein 1**  **Latent-transforming growth factor beta-binding protein 1**  **Minor histocompatibility protein HA-1;Minor histocompatibility antigen HA-1**  **Olfactomedin-like protein 1**  **Sushi domain-containing protein 2**  **Versican core protein**  **Plasmalemma vesicle-associated protein**  **EGF-containing fibulin-like extracellular matrix protein 2**  **Fibulin-5**  **C-type mannose receptor 2**  **ATP-dependent 6-phosphofructokinase, platelet type**  **Leucine-rich repeat-containing protein 32**  **Integrin alpha-3;Integrin alpha-3 heavy chain;Integrin alpha-3 light chain**  **Olfactomedin-like protein 3**  **EGF-containing fibulin-like extracellular matrix protein 1**  **Nephronectin**  **Latent-transforming growth factor beta-binding protein 4**  **Arf-GAP with coiled-coil, ANK repeat and PH domain-containing protein 1**  **Latent-transforming growth factor beta-binding protein 2**  **Angiopoietin-related protein 2**  **Glypican-4;Secreted glypican-4**  **Multiple epidermal growth factor-like domains protein 6**  **Carbohydrate sulfotransferase 14**  **Carbohydrate sulfotransferase 4**  **Cytochrome P450 3A7**  **TRAF3-interacting JNK-activating modulator**  **MAM domain-containing protein 2**  **Lysyl oxidase homolog 1**  **C-C motif chemokine 21**  **Hemicentin-1** | **LMNB1**  **SAMHD1**  **HSPG2**  **VIM**  **HLA-DRA**  **ANXA2;ANXA2P2**  **SLC35F6**  **KRT7**  **FLNA**  **FBN1**  **ATP8B1**  **PRELP**  **IGHA1**  **ANKRD44**  **EMILIN1**  **NID1**  **LAMA5**  **DBN1**  **AGRN**  **AQP1**  **ANGPTL6**  **LSP1**  **GPC6**  **VWA1**  **PFKFB2**  **S100A10**  **THY1**  **GZMK**  **COL12A1**  **SGCD**  **PTPRCAP**  **TACSTD2**  **SLC12A2**  **ALPK3**  **THSD4**  **LAMB1**  **AEBP1**  **LTBP1**  **HMHA1**  **OLFML1**  **SUSD2**  **VCAN**  **PLVAP**  **EFEMP2**  **FBLN5**  **MRC2**  **PFKP**  **LRRC32**  **ITGA3**  **OLFML3**  **EFEMP1**  **NPNT**  **LTBP4**  **ACAP1**  **LTBP2**  **ANGPTL2**  **GPC4**  **MEGF6**  **CHST14**  **CHST4**  **CYP3A7**  **TRAF3IP3**  **MAMDC2**  **LOXL1**  **CCL21**  **HMCN1** | **-0.325514191**  **-0.462798124**  **-0.50390747**  **-0.597414598**  **-0.667531104**  **-0.710340782**  **-0.776748348**  **-0.781359265**  **-0.800704438**  **-0.843312008**  **-0.886562435**  **-0.901958633**  **-0.933758897**  **-0.987806547**  **-1.083223662**  **-1.095191926**  **-1.120653093**  **-1.134704699**  **-1.136582121**  **-1.168804171**  **-1.176428191**  **-1.205863317**  **-1.226864156**  **-1.291283217**  **-1.306443812**  **-1.323607597**  **-1.356509703**  **-1.373863319**  **-1.380624344**  **-1.386763536**  **-1.483811151**  **-1.486393754**  **-1.51607909**  **-1.518452333**  **-1.564166937**  **-1.620447112**  **-1.632897167**  **-1.634442486**  **-1.635958541**  **-1.688184711**  **-1.692929572**  **-1.714176666**  **-1.828988667**  **-1.87620415**  **-1.895435501**  **-1.988695811**  **-2.032573294**  **-2.227139144**  **-2.293848495**  **-2.361814751**  **-2.362875753**  **-2.476766293**  **-2.503255355**  **-2.635702396**  **-2.720936565**  **-3.015896734**  **-3.194406621**  **-3.259064386**  **-3.353431249**  **-3.465618018**  **-3.509794551**  **-3.662808217**  **-4.47276799**  **-4.828010076**  **-5.38314313**  **-6.163304441** | **0.044**  **0.035**  **0.039**  **0.008**  **0.049**  **0.049**  **0.011**  **0.031**  **0.024**  **0.019**  **0.046**  **0.049**  **0.014**  **0.035**  **0.01**  **0.007**  **0.01**  **0.011**  **0.035**  **0.01**  **0.049**  **0.024**  **0.024**  **0.021**  **0.027**  **0.019**  **0.044**  **0.017**  **0.049**  **0.02**  **0.016**  **0.027**  **0.038**  **0.002**  **0.022**  **0.011**  **0.044**  **0.011**  **0.048**  **0.005**  **0.01**  **0.024**  **0.03**  **0.001**  **0.019**  **0.031**  **0.024**  **0.03**  **0.006**  **0.019**  **0.005**  **0.047**  **0.003**  **0.016**  **0.015**  **0.009**  **0.013**  **0.03**  **0.028**  **0.041**  **0.001**  **0.008**  **0.029**  **0.006**  **0.016**  **0.001** | **down-regulated**  **down-regulated**  **down-regulated**  **down-regulated**  **down-regulated**  **down-regulated**  **down-regulated**  **down-regulated**  **down-regulated**  **down-regulated**  **down-regulated**  **down-regulated**  **down-regulated**  **down-regulated**  **down-regulated**  **down-regulated**  **down-regulated**  **down-regulated**  **down-regulated**  **down-regulated**  **down-regulated**  **down-regulated**  **down-regulated**  **down-regulated**  **down-regulated**  **down-regulated**  **down-regulated**  **down-regulated**  **down-regulated**  **down-regulated**  **down-regulated**  **down-regulated**  **down-regulated**  **down-regulated**  **down-regulated**  **down-regulated**  **down-regulated**  **down-regulated**  **down-regulated**  **down-regulated**  **down-regulated**  **down-regulated**  **down-regulated**  **down-regulated**  **down-regulated**  **down-regulated**  **down-regulated**  **down-regulated**  **down-regulated**  **down-regulated**  **down-regulated**  **down-regulated**  **down-regulated**  **down-regulated**  **down-regulated**  **down-regulated**  **down-regulated**  **down-regulated**  **down-regulated**  **down-regulated**  **down-regulated**  **down-regulated**  **down-regulated**  **down-regulated**  **down-regulated**  **down-regulated** |
