# Supplementary material for: Use of proteomics to identify mechanisms of hepatocellular carcinoma with the CYP2D6*10 polymorphism and identification of ANGPTL6 as a new diagnostic and prognostic biomarker
Source: J Transl Med. 2021 Aug 19;19:359. doi: 10.1186/s12967-021-03038-3 (PMC8375140; doi:10.1186/s12967-021-03038-3)
Supplement: Supplementary file 4 — Additional file 4: Table S4. Compared with healthy subjects, the 17 upregulated and 71 downregulated DEPs in HCC groups based on proteomics analysis. [file 12967_2021_3038_MOESM4_ESM.docx]

Table S4 Compared with healthy subjects, the 17 upregulated and 71 downregulated DEPs in HCC groups based on proteomics analysis

| Proteins | Genes | logFC | P value | Group |
| --- | --- | --- | --- | --- |
| \| Melanotransferrin \| \| --- \| \| Angiotensin-converting enzyme;Angiotensin-converting enzyme, soluble form \| \| Beta-chimaerin \| \| GRAM domain-containing protein 4 \| \| Mitochondrial sodium/hydrogen exchanger 9B2 \| \| 6-phosphofructo-2-kinase/fructose-2,6-bisphosphatase 2;6-phosphofructo-2-kinase;Fructose-2,6-bisphosphatase \| \| Glycine dehydrogenase (decarboxylating), mitochondrial \| \| Serine protease hepsin;Serine protease hepsin non-catalytic chain;Serine protease hepsin catalytic chain \| \| Cytochrome P450 4V2 \| \| Integrin beta-3 \| \| Lethal(2) giant larvae protein homolog 2 \| \| Lanosterol synthase \| \| Acetyl-CoA carboxylase 2;Biotin carboxylase \| \| Dehydrogenase/reductase SDR family member 1 \| \| Phospholipid-transporting ATPase IG \| \| Peroxisomal targeting signal 1 receptor \| \| Lamin-B1 \| | MFI2  ACE  CHN2  GRAMD4  SLC9B2  PFKFB2  GLDC  HPN  CYP4V2  ITGB3  LLGL2  LSS  ACACB  DHRS1  ATP11C  PEX5  LMNB1 | -3.126938959  -1.477063056  -1.384889407  -1.273389879  -1.110364428  -1.097995877  -0.917482761  -0.872593073  -0.73295496  -0.691181975  -0.612948454  -0.498972041  -0.473835441  -0.432736434  -0.34618735  -0.305328456  -0.289712584 | 1.02591E-05  0.019093873  0.06528622  0.052575036  0.158904054  0.132836144  0.008208577  0.070959124  0.193085179  0.084277026  0.04235339  0.00134015  0.067091375  0.007531641  0.150439892  0.213416131  0.046523581 | down-regulated  down-regulated  down-regulated  down-regulated  down-regulated  down-regulated  down-regulated  down-regulated  down-regulated  down-regulated  down-regulated  down-regulated  down-regulated  down-regulated  down-regulated  down-regulated  down-regulated |
| NADH dehydrogenase [ubiquinone] 1 alpha subcomplex subunit 10, mitochondrial  Vimentin  Extended synaptotagmin-1  Basement membrane-specific heparan sulfate proteoglycan core protein;Endorepellin;LG3 peptide  Ig alpha-1 chain C region  Nidogen-1  Laminin subunit alpha-5  Filamin-A  Annexin A2;Putative annexin A2-like protein  Protein S100-A10  Deoxynucleoside triphosphate triphosphohydrolase SAMHD1  Prolargin  Exocyst complex component 3  ERO1-like protein beta  EMILIN-1  HLA class II histocompatibility antigen, DR alpha chain  Fibrillin-1  Agrin;Agrin N-terminal 110 kDa subunit;Agrin C-terminal 110 kDa subunit;Agrin C-terminal 90 kDa fragment;Agrin C-terminal 22 kDa fragment  Aquaporin-1  Glypican-6;Secreted glypican-6  Drebrin  Lymphocyte-specific protein 1  Thy-1 membrane glycoprotein  Olfactomedin-like protein 1  Laminin subunit beta-1  von Willebrand factor A domain-containing protein 1  Ataxin-2-like protein  Solute carrier family 35 member F6  Hemicentin-1  Leucine-rich repeat-containing protein 32  Collagen alpha-1(XII) chain  Serine/threonine-protein phosphatase 6 regulatory ankyrin repeat subunit B  Plasmalemma vesicle-associated protein  Phospholipid-transporting ATPase IC  Minor histocompatibility protein HA-1;Minor histocompatibility antigen HA-1  Keratin, type II cytoskeletal 7  TRAF3-interacting JNK-activating modulator  Arf-GAP with coiled-coil, ANK repeat and PH domain-containing protein 1  Adipocyte enhancer-binding protein 1  C-type mannose receptor 2  Integrin alpha-3;Integrin alpha-3 heavy chain;Integrin alpha-3 light chain  Thrombospondin type-1 domain-containing protein 4  Sushi domain-containing protein 2  ATP-dependent 6-phosphofructokinase, platelet type  Olfactomedin-like protein 3  Delta-sarcoglycan  Arf-GAP domain and FG repeat-containing protein 1  MAM domain-containing protein 2  Alpha-protein kinase 3  Carbohydrate sulfotransferase 14  Perilipin-4  Latent-transforming growth factor beta-binding protein 2  Granzyme K  Versican core protein  Glypican-4;Secreted glypican-4  Fibulin-5  Latent-transforming growth factor beta-binding protein 1  Multiple epidermal growth factor-like domains protein 6  Tumor-associated calcium signal transducer 2  Solute carrier family 12 member 2  Angiopoietin-related protein 2  Protein tyrosine phosphatase receptor type C-associated protein  Angiopoietin-related protein 6  Latent-transforming growth factor beta-binding protein 4  Nephronectin  C-C motif chemokine 21  Carbohydrate sulfotransferase 4  EGF-containing fibulin-like extracellular matrix protein 2  Lysyl oxidase homolog 1  Cytochrome P450 3A7  EGF-containing fibulin-like extracellular matrix protein 1 | NDUFA10  VIM  ESYT1  HSPG2  IGHA1  NID1  LAMA5  FLNA  ANXA2;ANXA2P2  S100A10  SAMHD1  PRELP  EXOC3  ERO1LB  EMILIN1  HLA-DRA  FBN1  AGRN  AQP1  GPC6  DBN1  LSP1  THY1  OLFML1  LAMB1  VWA1  ATXN2L  SLC35F6  HMCN1  LRRC32  COL12A1  ANKRD44  PLVAP  ATP8B1  HMHA1  KRT7  TRAF3IP3  ACAP1  AEBP1  MRC2  ITGA3  THSD4  SUSD2  PFKP  OLFML3  SGCD  AGFG1  MAMDC2  ALPK3  CHST14  PLIN4  LTBP2  GZMK  VCAN  GPC4  FBLN5  LTBP1  MEGF6  TACSTD2  SLC12A2  ANGPTL2  PTPRCAP  ANGPTL6  LTBP4  NPNT  CCL21  CHST4  EFEMP2  LOXL1  CYP3A7  EFEMP1 | 0.221596446  0.248883189  0.267224411  0.277062741  0.394612421  0.412345094  0.434403173  0.507402607  0.538956277  0.553899217  0.612929715  0.666671477  0.672097773  0.678698821  0.686331845  0.688264781  0.69932729  0.702856843  0.792628076  0.803272673  0.810259796  0.849974416  0.894321693  0.912282548  0.937429751  0.961699243  1.001986069  1.022158101  1.032014061  1.045082488  1.076604066  1.078846511  1.245039128  1.248057198  1.274688414  1.275825321  1.321995084  1.322524343  1.360006832  1.418336643  1.457130786  1.508934741  1.518627635  1.519057499  1.574246884  1.611085975  1.635886569  1.729912809  1.756382284  1.810388884  1.815295131  1.816220282  1.823302396  1.83197478  1.921729738  2.213745533  2.277269048  2.389946077  2.399467204  2.573864454  2.589142042  2.59439905  2.601698683  2.697427097  2.699425724  2.721041439  2.868985292  3.328116639  3.524430031  3.671712178  3.736926274 | 0.083992915  0.209120141  0.048497091  0.172200015  0.193897468  0.211045962  0.213416131  0.066696618  0.068793889  0.124928596  0.00331997  0.051352859  0.209580637  0.162518995  0.015117393  0.010401421  0.008795977  0.070071509  0.014301393  0.049156004  0.093886613  0.113081646  0.059367113  0.179686767  0.100555338  0.128082391  0.202029203  0.220689852  0.13915408  0.154123165  0.017799135  0.154123165  0.223234074  0.034065263  0.128623645  0.000605951  0.172918631  0.025257602  0.076998925  0.059381844  0.067313117  0.048497091  0.00594273  0.032300792  0.086788181  0.085521944  0.115354065  0.046061096  0.011223969  0.076998925  0.003180559  0.024425288  0.034069013  0.045182026  0.024425288  0.007673234  0.000148526  0.001433056  0.010756438  0.000816087  0.003269299  0.032883401  1.07912E-10  0.001089469  0.001256335  0.016120653  0.003151525  0.000174589  0.00021342  0.003180559  0.000244287 | up-regulated  up-regulated  up-regulated  up-regulated  up-regulated  up-regulated  up-regulated  up-regulated  up-regulated  up-regulated  up-regulated  up-regulated  up-regulated  up-regulated  up-regulated  up-regulated  up-regulated  up-regulated  up-regulated  up-regulated  up-regulated  up-regulated  up-regulated  up-regulated  up-regulated  up-regulated  up-regulated  up-regulated  up-regulated  up-regulated  up-regulated  up-regulated  up-regulated  up-regulated  up-regulated  up-regulated  up-regulated  up-regulated  up-regulated  up-regulated  up-regulated  up-regulated  up-regulated  up-regulated  up-regulated  up-regulated  up-regulated  up-regulated  up-regulated  up-regulated  up-regulated  up-regulated  up-regulated  up-regulated  up-regulated  up-regulated  up-regulated  up-regulated  up-regulated  up-regulated  up-regulated  up-regulated  up-regulated  up-regulated  up-regulated  up-regulated  up-regulated  up-regulated  up-regulated  up-regulated  up-regulated |
