# Supplementary material for: Use of proteomics to identify mechanisms of hepatocellular carcinoma with the CYP2D6*10 polymorphism and identification of ANGPTL6 as a new diagnostic and prognostic biomarker
Source: J Transl Med. 2021 Aug 19;19:359. doi: 10.1186/s12967-021-03038-3 (PMC8375140; doi:10.1186/s12967-021-03038-3)
Supplement: Supplementary file 5 — Additional file 5: Table S5. DEPs with the area under the ROC curve (AUC) > 7.0. [file 12967_2021_3038_MOESM5_ESM.docx]

**Table S5 DEPs with the area under the ROC curve (AUC) > 7.0**

| Protein.names | Gene.names | P Value | AUC | logFC |
| --- | --- | --- | --- | --- |
| \| Angiopoietin-related protein 6 \| \| --- \| \| Latent-transforming growth factor beta-binding protein 1 \| \| EGF-containing fibulin-like extracellular matrix protein 2 \| \| EGF-containing fibulin-like extracellular matrix protein 1 \| \| Keratin, type II cytoskeletal 7 \| \| Fibulin-5 \| \| Sushi domain-containing protein 2 \| \| Latent-transforming growth factor beta-binding protein 4 \| \| Nephronectin \| \| Lysyl oxidase homolog 1 \| \| Solute carrier family 12 member 2 \| \| Delta-sarcoglycan \| \| Perilipin-4 \| \| Cytochrome P450 3A7 \| \| Multiple epidermal growth factor-like domains protein 6 \| \| Fibrillin-1 \| \| Deoxynucleoside triphosphate triphosphohydrolase SAMHD1 \| \| Carbohydrate sulfotransferase 4 \| \| HLA class II histocompatibility antigen, DR alpha chain \| \| Alpha-protein kinase 3 \| \| Angiopoietin-related protein 2 \| \| Aquaporin-1 \| \| C-type mannose receptor 2 \| \| Collagen alpha-1(XII) chain \| \| EMILIN-1 \| \| ATP-dependent 6-phosphofructokinase, platelet type \| \| Serine/threonine-protein phosphatase 6 regulatory ankyrin repeat subunit B \| \| Thy-1 membrane glycoprotein \| \| Granzyme K \| \| Prolargin \| | \| ANGPTL6 \| \| --- \| \| LTBP1 \| \| EFEMP2 \| \| EFEMP1 \| \| KRT7 \| \| FBLN5 \| \| SUSD2 \| \| LTBP4 \| \| NPNT \| \| LOXL1 \| \| SLC12A2 \| \| SGCD \| \| PLIN4 \| \| CYP3A7 \| \| MEGF6 \| \| FBN1 \| \| SAMHD1 \| \| CHST4 \| \| HLA-DRA \| \| ALPK3 \| \| ANGPTL2 \| \| AQP1 \| \| MRC2 \| \| COL12A1 \| \| EMILIN1 \| \| PFKP \| \| ANKRD44 \| \| THY1 \| \| GZMK \| \| PRELP \| | \| 0.049 \| \| --- \| \| 0.011 \| \| 0.001 \| \| 0.005 \| \| 0.031 \| \| 0.019 \| \| 0.01 \| \| 0.003 \| \| 0.047 \| \| 0.006 \| \| 0.038 \| \| 0.02 \| \| 0.035 \| \| 0.001 \| \| 0.03 \| \| 0.019 \| \| 0.035 \| \| 0.041 \| \| 0.049 \| \| 0.002 \| \| 0.009 \| \| 0.01 \| \| 0.031 \| \| 0.049 \| \| 0.01 \| \| 0.024 \| \| 0.035 \| \| 0.044 \| \| 0.017 \| \| 0.049 \| | \| 0.900193 \| \| --- \| \| 0.83028 \| \| 0.814127 \| \| 0.812922 \| \| 0.804243 \| \| 0.790743 \| \| 0.785921 \| \| 0.777242 \| \| 0.766635 \| \| 0.763742 \| \| 0.7635 \| \| 0.761331 \| \| 0.75217 \| \| 0.750723 \| \| 0.748071 \| \| 0.739634 \| \| 0.736258 \| \| 0.729026 \| \| 0.728062 \| \| 0.722276 \| \| 0.71866 \| \| 0.717936 \| \| 0.717936 \| \| 0.716972 \| \| 0.715526 \| \| 0.706847 \| \| 0.704436 \| \| 0.702507 \| \| 0.702266 \| \| 0.700096 \| | \| 2.601699 \| \| --- \| \| 2.277269 \| \| 3.328117 \| \| 3.736926 \| \| 1.275825 \| \| 2.213746 \| \| 1.518628 \| \| 2.697427 \| \| 2.699426 \| \| 3.52443 \| \| 2.573864 \| \| 1.611086 \| \| 1.815295 \| \| 3.671712 \| \| 2.389946 \| \| 0.699327 \| \| 0.61293 \| \| 2.868985 \| \| 0.688265 \| \| 1.756382 \| \| 2.589142 \| \| 0.792628 \| \| 1.418337 \| \| 1.076604 \| \| 0.686332 \| \| 1.519057 \| \| 1.078847 \| \| 0.894322 \| \| 1.823302 \| \| 0.666671 \| |
