# Supplementary figures and images for: Use of proteomics to identify mechanisms of hepatocellular carcinoma with the CYP2D6*10 polymorphism and identification of ANGPTL6 as a new diagnostic and prognostic biomarker
Source: J Transl Med. 2021 Aug 19;19:359. doi: 10.1186/s12967-021-03038-3 (PMC8375140; doi:10.1186/s12967-021-03038-3)

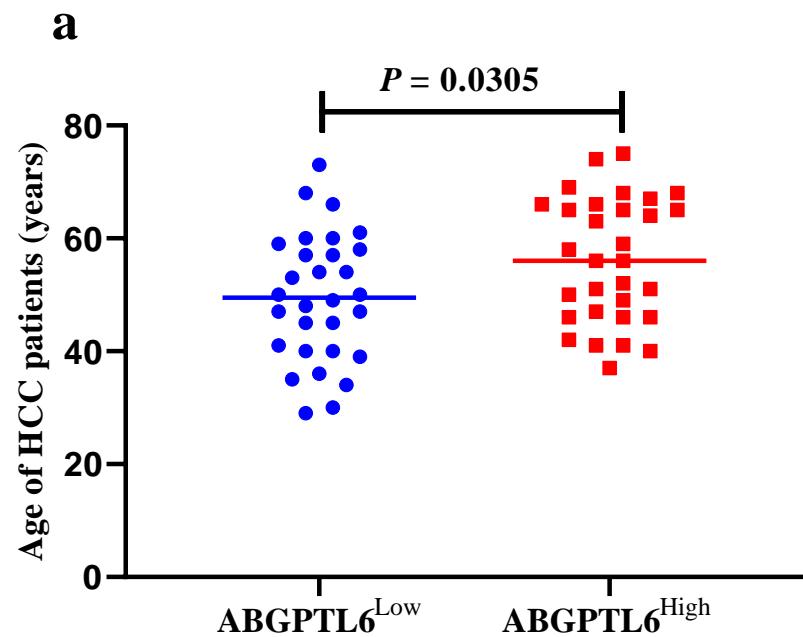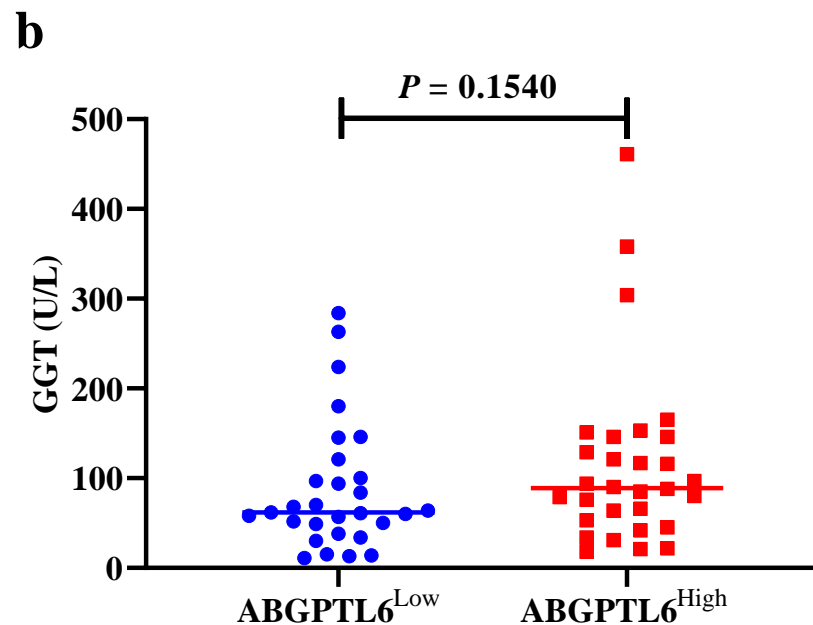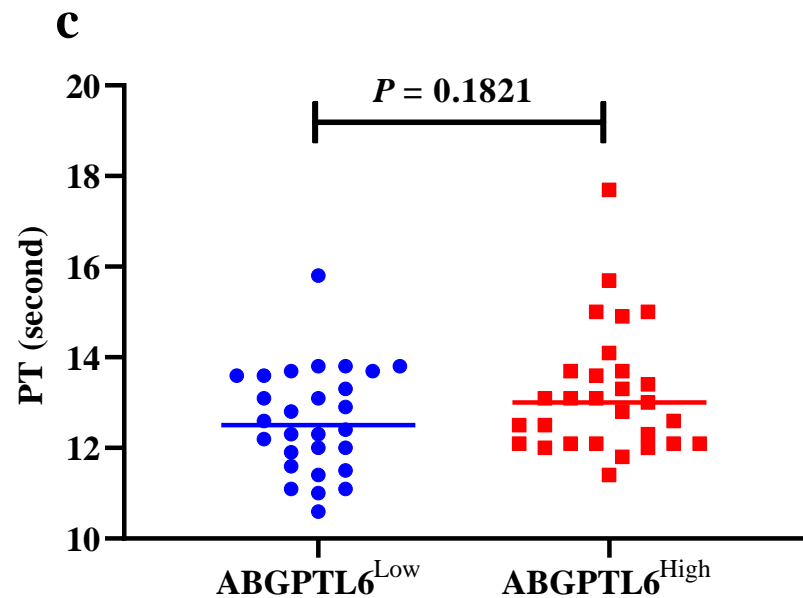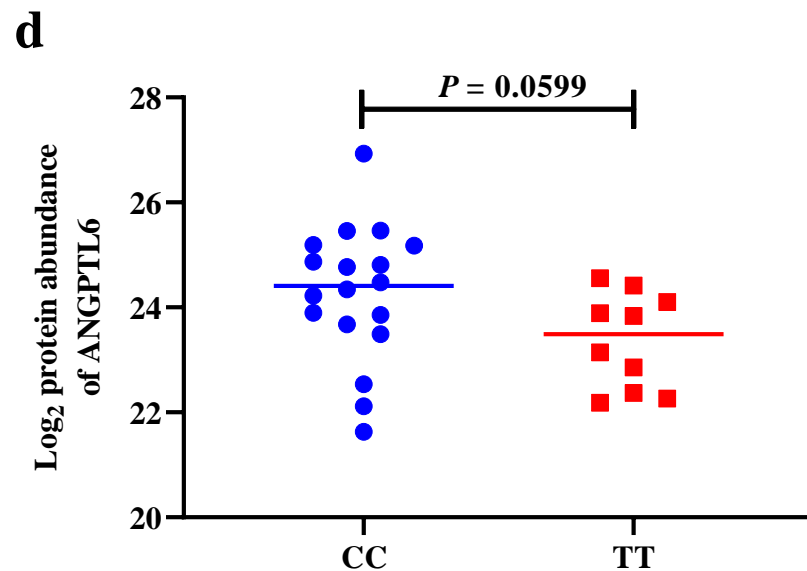

Supplement: Supplementary file 6 — Additional file 6: Figure S1. ANGPTL6 has clinical significance. a High ANGPTL6 expression was more common in the elderly group. Serum levels of GGT (b) and PT (c) show an increasing trend in the high ANGPTL6 groups. d ANGPTL6 in TT and CC group. HCC, hepatocellular carcinoma. GGT, gamma-glutamyl transpeptidase. PT, prothrombin time. [file 12967_2021_3038_MOESM6_ESM.pdf]
